# Assessment of the Empact of IGC‐AD1 on Hepatic Biomarkers in Alzheimer’s Disease: Insights from a Phase 1 Trial

**DOI:** 10.1002/alz.095744

**Published:** 2025-01-09

**Authors:** Diego Rodriguez‐Soacha, Andres Cañon, Laura Tatiana Sánchez, Jagadeesh S Rao, Laura Delgado‐Murillo, Margarita Venegas, Maria Alejandra Tangarife, Maria Juanita Arbelaez, Evelyn Gutiérrez, Saadia Shahnawaz, Varduhi Ghazaryan, William Julio De Jesus, Claudia Grimaldi, Elliot Hong, Ram Mukunda

**Affiliations:** ^1^ IGC Pharma, Bogotá, Bogotá D.C. Colombia; ^2^ IGC Pharma, Potomac, MD USA; ^3^ Santa Cruz Behavioral, Bayamon, PR USA; ^4^ UTHealth Houston McGovern Medical School, Houston, TX USA

## Abstract

**Background:**

IGC‐AD1 comprises of Tetrahydrocannabinol (“THC”) and melatonin. The two active pharmaceuticals are known for their neuroprotective properties. In this analysis we studied multiple dosing of IGC‐AD1 in Alzheimer’s (“AD”) populations vulnerable to hepatic complications. We present the impact of three different doses on hepatic functions, offering insights into dosage‐dependent safety and potential therapeutic implications.

**Method:**

Thirteen Puerto Rican AD patients (mean age: 80.18±6.22 years, 70% women) participated in this three‐cohort trial to assess the effects of IGC‐AD1. Cohorts 1, 2 and 3 received 1 mL once, twice, or thrice daily, respectively, for 14 days (“EOT”) with ∼4‐day washout period between cohorts. Key hepatic and renal biomarkers measured at baseline and EOT included AST, ALT, ALP, total and direct bilirubin.

**Result:**

Findings showed a larger increase in the mean difference of ALTI in the placebo group compared to active in cohorts 2 and 3 (Cohort‐1, Active; = ‐2.11 ± 6.01, Placebo = ‐0.5 ± 0.71; Cohort‐2, Active = ‐2.25 ± 4,79, Placebo = ‐8.0; Cohort‐3, Active; = ‐3.5 ± 9.73, Placebo = ‐7.0 ± 8.49). ALP levels decreased for both treatments in cohort 1 but increased for cohorts 2 and 3 (ALP Cohort‐1, Active; = 2.89 ± 4.34, Placebo = 3.5 ± 7.78; Cohort‐2, Active = ‐0.6± 18.15, Placebo = ‐5.0; Cohort‐3, Active; = ‐2.8 ±5.57, Placebo = ‐3.0 ± 7.07). Indirect bilirubin levels increased in the active groups for cohort 3, no tendency was observed for placebos (Indirect bilirubin Cohort‐1, Active; = 0.02 ± 0.05, Placebo = 0.05 ± 0.03; Cohort‐2, Active = 0.07 ± 0.07, Placebo = ‐0.03; Cohort‐3, Active; = ‐0.04 ± 0.14, Placebo = 0.07). All other biomarkers were within expected ranges, supporting the treatment’s safety profile.

**Conclusion:**

Based on this safety trial, the findings suggest that the treatment with THC and melatonin does not exert adverse effects on liver function in patients with AD. Overall, the treatment demonstrated good tolerability, with no significant differences in hepatic biomarkers compared to placebo, supporting its potential safety in elderly patients with AD. Future research should continue to monitor these biomarkers in larger, more diverse populations to confirm these findings and assess long‐term safety and efficacy.

